# FLASH effect is diminished by daily fractionation of electron RT in mouse skin

**DOI:** 10.1088/1361-6560/ae205e

**Published:** 2025-11-26

**Authors:** Aleksandra Ilina, William S Thomas, Xu Cao, Matthew S Reed, Kendall Jarvis, Albert van der Kogel, Nathaniel van Asselt, Wesley S Culberson, Brian W Pogue

**Affiliations:** 1 University of Wisconsin, Madison, WI, United States of America; 2 Dartmouth College, Hanover, NH, United States of America

**Keywords:** FLASH, fractionation, radiotherapy, UHDR

## Abstract

*Objective.* While FLASH radiotherapy is known to reduce skin damage *in vivo* from ultra-high dose rate (UHDR) irradiation relative to conventional dose rates (CDR), it is not clear whether this sparing is preserved when delivered as fractionated. This study was designed to directly assess whether three daily fractions would maintain the sparing effects in murine leg models and preserve the murine skin sparing with single fraction treatments. *Approach.* C57Bl/6j female mice were irradiated with 9 MeV UHDR and CDR beams from a FLASH-capable Mobetron system, in a dose escalation study with doses ranging from single dose 22–30 Gy in a single fraction to three daily fractions of 10–16 Gy. The biological responses were scored by a visual skin damage response rubric using up to 5 blinded observers, and a leg contracture assay as a secondary measure of damage. *Main results.* There was a monotonic dose response in all treatment groups with irradiation dose, with skin damage onset at 9–10 d. In the single dose group a significant FLASH sparing was seen with a FLASH modifying factor (FMF) of approximately 0.73. Similarly in the single dose groups there were significant leg contracture differences between UHDR and CDR groups after 12–15 d. In comparison, there was no significant skin damage sparing between UHDR and CDR in the three daily fraction dose groups, and reduced sparing in the leg contracture assay. *Significance.* The results of this murine study show a significant reduction of the FLASH effect when the dose is split into three fractions of 10–16 Gy each, whereas there were substantial FLASH sparing effects noted for the single fractions of 22–30 Gy, showing a FMF of ∼0.73. These observations provide the data needed to optimize FLASH sparing experiments in further translational studies.

## 1. Introduction

The ‘FLASH effect’ comprising of normal tissue sparing in radiation therapy (RT) with equivalent tumor control when radiation is delivered at ultra-high dose rate (UHDR) has created significant interest in the RT community since the key publications (Favaudon *et al* 2014, Montay-Gruel *et al* 2017, Bourhis *et al* 2019, Vozenin *et al* 2019). Recently many translational studies (Konradsson *et al* 2021, Velalopoulou *et al* 2021, Rohrer Bley *et al* 2022, Bohlen *et al* 2022a) have been completed to assess efficacy and optimization of this effect, and initial phase once clinical trials have started (Mascia *et al* 2023, Daugherty *et al* 2024, Kinj *et al* 2024, Goncalves Jorge *et al* 2025). One of the most important questions in UHDR FLASH is whether it is possible to deliver treatments in a fractionated manner while preserving the effect, to closely match what is feasible in clinical radiotherapy. This was the core motivation for this work.

There are two primary dosimetric factors that determine the FLASH effect, namely the dose rate) and the total dose delivered. The mean dose rate for maximal FLASH effect is thought to be >40 Gy s^−1^ (Montay-Gruel *et al* 2017, Sorensen *et al* 2024) although the exact threshold is still under study and may be dependent on other factors such as peak dose rate and temporal pulse train and the tissue type. The total dose required to see a FLASH effect varies with the tissue type irradiated and the assay to measure an effect (Bohlen *et al* 2022b), with the lowest total dose values required for inducing a FLASH effect appearing in brain function, near 8 Gy in a single delivery (Montay-Gruel *et al* 2017, Bohlen *et al* 2022b). Previous *in vivo* studies of skin damage indicate that a total dose of ∼20 Gy is required to see a FLASH effect (Bohlen *et al* 2022b).

It is largely accepted that high doses per fraction are required with singular dose delivery (Bohlen *et al* 2022a), but actually the dose per fraction to achieve FLASH has not been appropriately quantified with a standard approach to daily fractionation or even hypofractionation, except for limited studies (Verginadis *et al* 2025) with others focused primarily on neurocognitive sparing (Montay-Gruel *et al* 2021, Alaghband *et al* 2023, Limoli *et al* 2023). Studies in FLASH skin response showed that a split dose delivery, with ∼2 min between equal doses, reduced the magnitude of skin sparing effect (Mascia *et al* 2024, Sorensen *et al* 2024). With these developments in mind, a key next step is to determine if fractionated delivery over days, of the type commonly delivered in a hypofractionated treatment, lowers the FLASH effect, or if there is a way to achieve this in early translational studies. In the studies reported here, mouse skin response was tested to compare single dose deliveries to a 3 fractionation scheme with the goal of using the results to inform translation to larger animal or human studies.

Quantifying radiation effects in normal skin via visual assessment has been established as a robust assay in its timeline, repeatability, and translation. Specifically: (1) early vascular damage is expressed as erythema (2) the epithelial desquamation response is directly related to the depletion and subsequent regeneration of basal stem cells (3) it is a low-risk organ and already a primary assay in veterinary and human translational studies, and (4) nearly all radiation treatments implicitly involve irradiation of the skin. Due to these reasons skin response to irradiation has been extensively studied, and measures include both severity and timeline to recovery (Pottern 1985). Most pre-clinical studies evaluate acute damage based primarily on erythema and dry/moist desquamation and an estimate of the surface of the irradiated area showing these types of damage (Singers Sorensen *et al* 2022, Duval *et al* 2023, Zhang *et al* 2023, Rudigkeit *et al* 2024). However, it is important to recognize that the choice of the scoring methodology can affect the dose at which optimum FLASH sparing is seen. In this work, the response scoring system was carefully chosen to quantify the more subtle responses seen, such that if applied to further translational work it would lead to appropriately safe dose optimization.

This study design compared acute skin effects for three subsequent, daily fractions to single delivery of conventional dose rate (CDR) and UHDR irradiations). Doses were selected based on biologically effective dose (BED). BED is calculated using α/β values derived from the LQ model which are based solely on CDR datasets. A direct comparison of UHDR doses in fractions cannot occur under traditionally defined BED due to a lack of specific α/β values and knowledge of exactly how dose rate impacts BED on a tissue dependent level (Bohlen *et al* 2022a). The 3 fraction doses that were adjusted to single fraction doses were chosen as a basis for translation to dose escalation veterinary trials with 2 Gy increments. The lower dose bound in this study was based on single dose values established based on other murine skin studies complied by Bohlen *et al* to a point where a FLASH effect can be seen (>20 Gy) (2022b). The BED was additionally chosen to include an effect of fractionation as compared to simply matching total doses in this study (Bohlen *et al* 2022b). A previously validated rubric for scoring skin damage in mice was used (Velalopoulou *et al* 2021) with blinded evaluators, to quantify acute skin damage over 4 weeks. Additionally, unblinded assessment of acute leg contraction occurred alongside this as a secondary measure of skin damage.

## 2. Methods

### 2.1. Radiation delivery and dosimetry

A Mobetron (IntraOp, Sunnyvale, CA) electron linac with UHDR capabilities was used for both CDR and UHDR deliveries. A 10 cm field size was chosen to minimize penumbra effects of field shaping and the right hind leg of the mice was restrained within the field to keep a consistent dose across the mouse limb. Positioning was verified via film every day of irradiation.

Beam parameters for CDR were 9 MeV electrons with a beam structure of 30 Hz, pulse width of 1.2 *μ*s, and an average dose rate of 0.17 Gy s^−1^. Beam parameters for UHDR were 9 MeV electrons with a beam structure of 120 Hz, 2 *μ*s pulse width resulting in an average dose rate of 95–100 Gy s^−1^. Beam dose delivery was recorded via EBTXD Gafchromic film (Ashland, Bridgewater, NJ) (Del Sarto *et al* 2025). Film was placed onto a plastic holder to ensure identical film orientation for scanning pre and post radiation on an Epson expression 10 000 XL (Epson, Los Alamitos, CA) with changes in optical density compared to calibration films and known optical density filters. This film accuracy is expected to be ∼3% based upon film used for calibration of the Mobetron. Doses for each delivery group are reported in table 1 and measured as absorbed dose to water. To validate these delivered doses direct online measurement was used both during treatment, as well as in day-to-day machine monitoring, via a W2 Scintillator (Standard Imaging, Middleton, WI) and an Advanced Markus (PTW, Freiburg, Germany) ion chamber. Small modifications in pulse width were made to adjust doses to match desired values in fractionated datasets in the UHDR 3 × 16 Gy deliveries where pulse width was increased by 0.03 *μ*s respectively. This kept the instantaneous dose rate consistent between various deliveries while achieving desired dose delivery.

**Table 1.** Dose groups used in terms of number of daily fractions at each dose level, for mouse groups, with average dose per fraction. Dose reported is average with ± std. dev. as measured during treatments. Dose per pulse (DPP) was modulated via a fine tune of the pulse width modulator to reach the desired total dose level and denoted by a * for 0.03 *μ*s pulse length adjustment to achieve desired doses.

Dose group	BED (Gy)	# Mice	Dose rate group	Dose per fraction (Gy)	# Pulses	Dose per pulse (Gy)
1 × 22 Gy	—	5	UHDR	23.0 ± 0.4	29	0.79
1 × 22 Gy	70	5	CDR	22.1 ± 0.6	3.8 × 10^3^	5.7 × 10^−3^
1 × 24 Gy	—	5	UHDR	23.8 ± 0.7	30	0.79
1 × 24 Gy	82	5	CDR	24.0 ± 0.2	4.2 × 10^3^	5.7 × 10^−3^
1 × 30 Gy	—	5	UHDR	30.0 ± 0.6	38	0.79
1 × 30 Gy	120	5	CDR	30.4 ± 0.6	5.4 × 10^3^	5.7 × 10^−3^
3 × 10 Gy	—	5	UHDR	10.2 ± 0.2	13	0.78
3 × 10 Gy	60	5	CDR	9.9 ± 0.5	1.7 × 10^3^	5.7 × 10^−3^
3 × 12 Gy	—	4	UHDR	11.9 ± 0.2	15	0.79
3 × 12 Gy	79	5	CDR	12.2 ± 0.3	2.2 × 10^3^	5.7 × 10^−3^
3 × 16 Gy	—	7	UHDR	15.8 ± 0.6	19	0.83 *
3 × 16 Gy	125	7	CDR	16.0 ± 0.4	2.8 × 10^3^	5.7 × 10^−3^

### 2.2. Mouse irradiation

Animal Experiments were approved by the University of Wisconsin-Madison Institutional Animal care and Use Committee. C57Bl/6j female mice (Envigo RMS (Allison Pointe, Indianapolis, IN) were used at 8 weeks old, and housed in the university vivarium throughout study, except for the days of irradiation. This study adhered to ARRIVE guidelines for animal research. The right hind legs of the mice were shaved 1 d before irradiation to help with visual assessment of radiation-induced skin response.

A total of 80 mice were included in all experiments. The mice were separated in groups of 5–7 mice per dose group with group 3 × 12 having only 4 mice due to one mouse not surviving to followup (table 1). Two mice had to be excluded from the study due to technical problems during the dose delivery. Prescribed doses were chosen corresponding to BED values found in skin for both murine skin with alpha/beta ratio of ∼10 (Moulder and Fischer 1976, Fowler 1983). This resulted in single fraction doses of 22, 24, and 30 Gy alongside 3 fraction doses of 10, 12, and 16 Gy respectively these are displayed in table 1. The initial UHDR group of 1 × 22 Gy was found to have received 1 × 23 Gy after analysis of the Film used to track dosimetry, this was adjusted for the dosimetry of future groups. In the case of the fractionated dose the deliveries occurred over three consecutive days to mimic clinical RT methods, which can be done consecutively or every other day.

**Figure 1.**
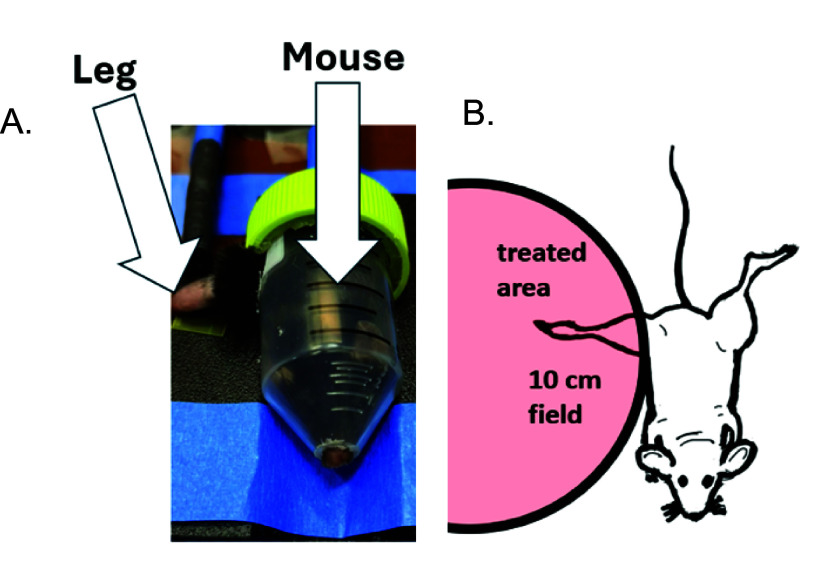
(A). Photograph of the mouse inside the immobilization holder, and (B). The leg geometry in the irradiation field.

During irradiation the mice were restrained inside a shortened 50 ml Eppendorf tube with breathing hole in one end and another for the tail to assist in immobilization, with a lateral hole for one leg to extend out from, seen in figure 1. This holder was used to avoid the use of anesthesia, which has potential to alter the radiation response and potentially suppress the FLASH effect (Tavakkoli *et al* 2023). Mice were arranged on the edge of a 10 cm circular radiation field with the leg positioned into the field edge to minimize penumbral effects. The mouse tube was immobilized in a 3D printed jig to restrain movement and keep positioning within the field identical between successive mice, with film used to position the jig to ensure accurate, homogenous dose delivery. The right hind leg of each mouse was extended through the hole in the side of the tube and taped lightly to the platform, as distally as possible to ensure the limb stayed in the correct irradiation position during treatment. A plastic insert was used for film collimation to minimize photon contamination.

### 2.3. Radiation-induced damage response assays

The primary radiation response assay was acute skin toxicity, quantified through interpretation of photographs of the animal limbs, taken every 2–3 d, for a total of 4 weeks post irradiation. If mice exhibited distress they received ketoprofen (Bimedia Inc., (Le Suere, MN) following our scoring schedule. These were quantified using a previously established scoring scale (Velalopoulou *et al* 2021). The scoring table is shown in table 2. This ten point scoring system was used to focus on sensitivity of our assay. The blind scoring occurred as follows: Images of the mice were taken in a diffuse light environment, and, images were cropped to allow for blinded reading of each mouse hind leg. The images were randomized per day of evaluation and each of 4–5 readers assigned a score at the end of the evaluation period. Readers were trained through discussion with example images, and the results from all were then averaged per mice, and then per dose group.

**Table 2.** Skin damage scoring system with descriptors (Velalopoulou *et al* 2021).

Score	DESCRIPTION OF SKIN RESPONSE OBSERVED AT EACH LEVEL
0.5	A chance there is a difference from healthy skin
0.75	Definite but slight abnormality
1.0	Definite abnormality with skin reddening
1.25	Severe reddening with white scales and/or puffiness
1.5	Moist breakdown in one very small area, with scaly or crusty appearance
1.75	Moist desquamation in moderate areas
2.0	Skin breakdown in large areas, possibly moist
2.5	Breakdown in large areas of skin with definite moist exudate
3.0	Breakdown in most skin with definite moist exudate
3.5	Complete breakdown of limb, stuck to body

A secondary assay of radiation response was carried out using a measurement of leg contracture. Leg contracture was conducted via pulling of the hind limbs from a suspended position, with the numeric scoring system used shown in table 3. Each of these latter assays occurred on the same days upon which mice were being photographed. This was unblinded due to the nature of physically performing the measurements on the designated mice, while the imaging benefitted from blinding in the processing of the images. If a mouse exhibited severe skin damage (∼3.0) the leg was very immobile and clearly fused to the body. In these cases no contraction was performed and the evaluation given a score of 5.

**Table 3.** Scoring system for acute leg contracture radiation response.

Score	DESCRIPTION OF CONTRACTURE AT THIS LEVEL
0	Full ability to retract
1	Minor issue with retraction
2	Ability to retract only halfway
3	Major issue with retraction
4	Almost no retraction, limb is not fused
5	No contraction limb is fused in its socket and immobile.

#### 2.3.1. Statistical analysis

Data accumulated were for skin response assay (0–3.5 scale, table 2), as acquired from 4–5 blinded readers for every mouse for all days, averaging the results together for each day. The secondary data accumulated of leg contracture response (0–5 scale, table 3) was tabulated from two observers in a non-blinded manner. Statistical analysis was performed for differences between treatment groups based upon these two data sets, with the differences between groups being assessed by a 2-tailed student’s t-test, using a *P*-value<0.05 as the threshold for significance.

## 3. Results

**Figure 2.**
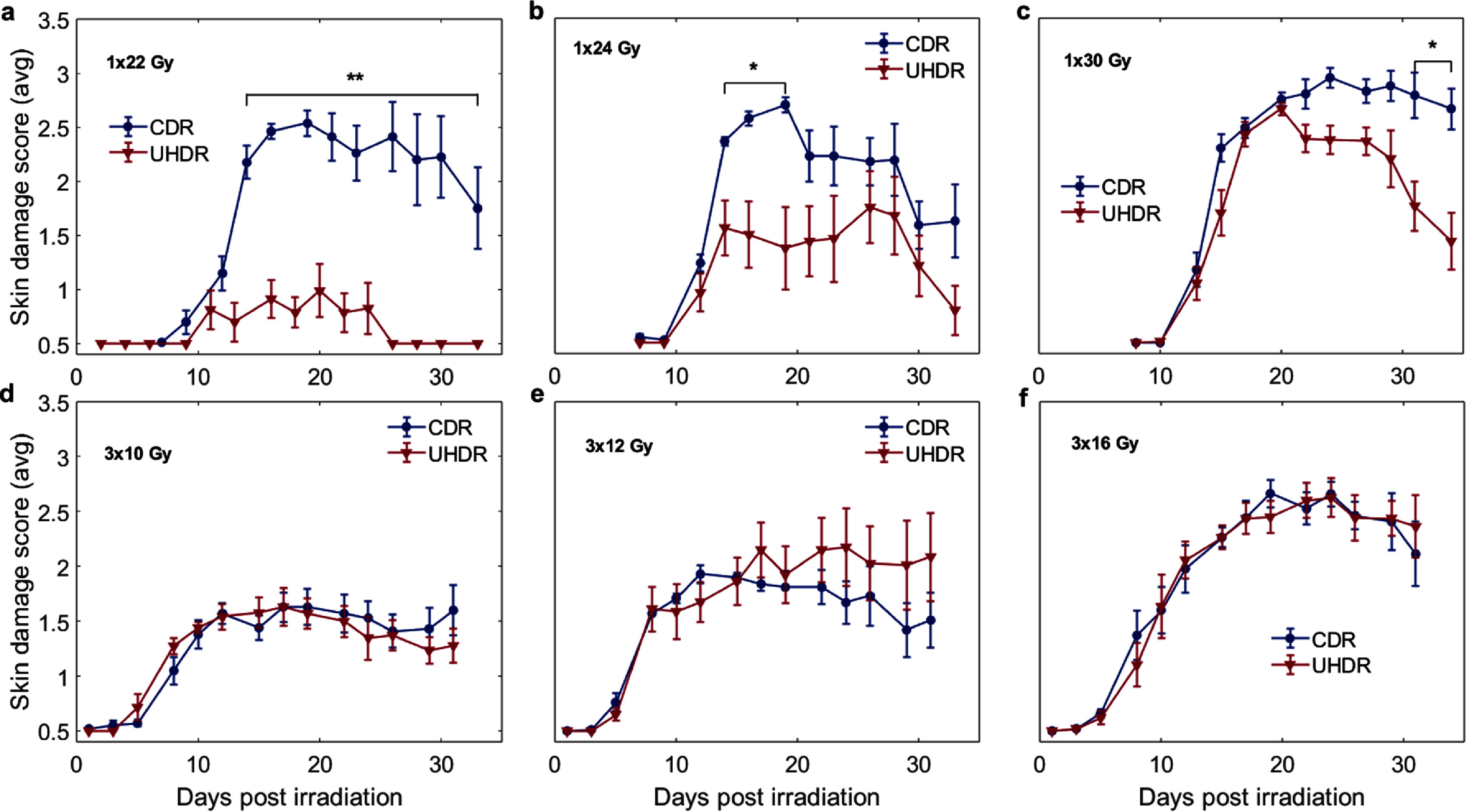
Skin radiation damage score values are shown for single dose treatment groups of increasing total dose (a)–(c) and then for the three fraction delivery groups with increasing dose (d)–(f). The top and bottom rows were designed to have roughly the same BED. Statistically significant differences between skin score of CDR and UHDR were only seen in the single fraction groups (a)–(c). Error bars are plotted according to standard error. Significant differences between CDR and UHDR groups are denoted by * * *P* < 0.05, ** *P* < 0.01.

In the case of all single dose deliveries (figures 2(a)–(c)) a FLASH effect was observed, as seen by a significant lowering of the skin response between the UHDR group as compared to the CDR group. The difference between UHDR and CDR was significant (*P* < 0.05), and visible in the graph, demonstrating a skin-sparing effect, which was the most noticeable for the lower doses of 22 & 24 Gy, after 12 d. Sparing was still observed in the 30 Gy single dose delivery when both CDR and UHDR reached the same magnitude of damage ∼3.0, but this diverged as the UHDR group began to heal (*P* < 0.05), this healing is well known in response, and generally attributed to basal stem cells surviving the radiation damage (Joiner 2018). The lack of healing being observed at the highest dose levels implies that basal stems cells were largely depleted due to the radiation and so the skin had limited ability to repair the damage.

The fractionated UHDR irradiation compared to fractionated CDR (figures 2(d)–(f)) show no significant differences between groups, indicating that the FLASH effect was not present. Clear hallmarks of skin radiobiology can be seen as a result of these experiments in both the timeline and dose response. We observed that the timeline of skin damage development for both CDR and UHDR did not differ from one another within the same dose group and remained consistent with published data on damage developing after 9 d with peak damage occurring at ∼15 d post irradiation (Pottern 1985, Sorensen *et al* 2024). Furthermore there is a clear, monotonic dose response observed in all treatment groups with the mean skin damage score increasing with delivered dose for both single fraction and fractionated deliveries, validating the stability of this assay.

**Figure 3.**
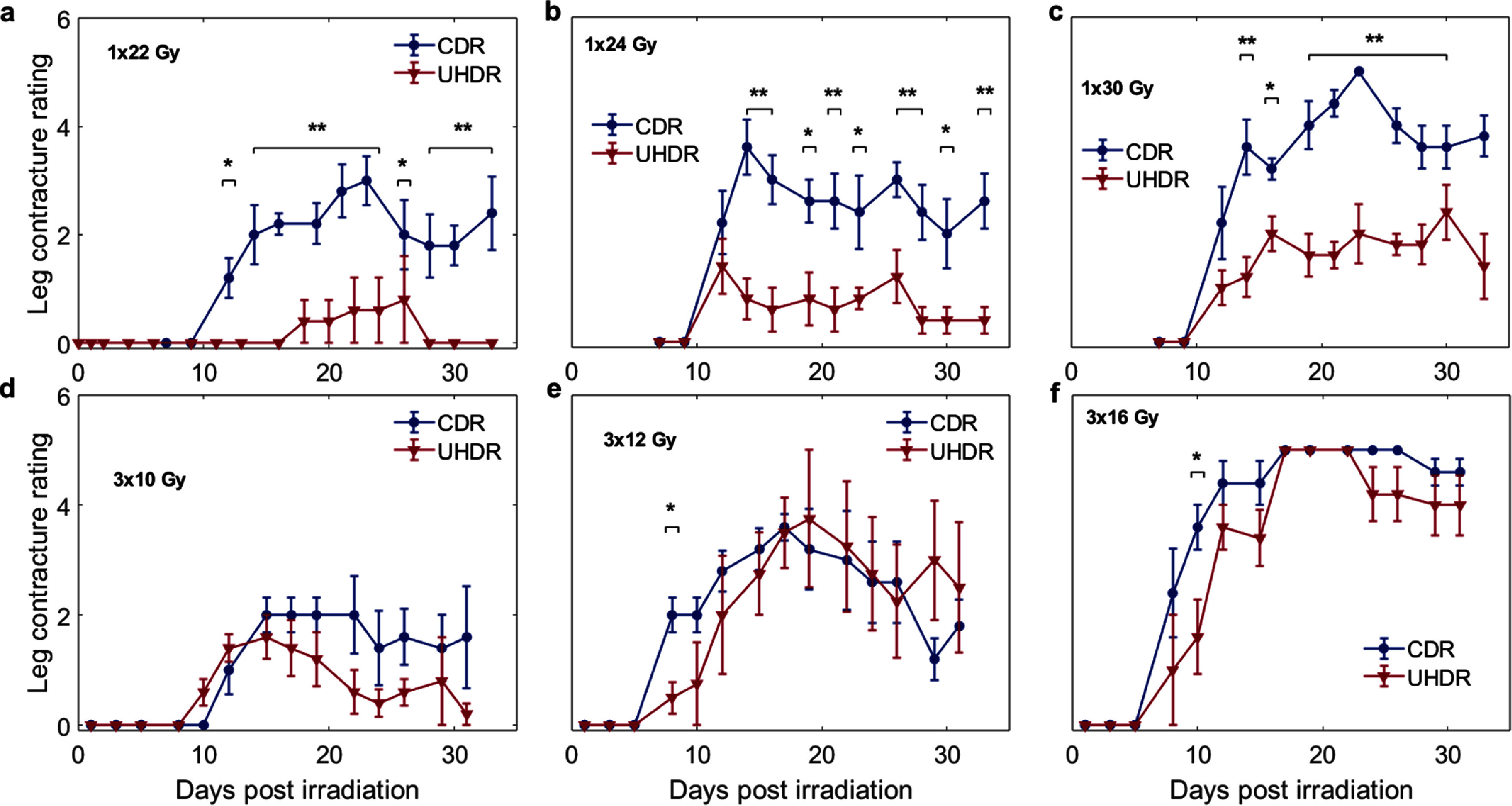
Leg contracture assay results shown for single dose treatment groups of increasing total dose (a)–(c) and for the three fraction groups (d)–(f) with similar increasing dose. The top and bottom rows were designed to have roughly parallel BED values. Error bars are plotted according to standard error. Significant differences between CDR and UHDR groups are denoted by * *P* < 0.05, ** *P* < 0.01.

The secondary assay measured leg contracture by comparing the treated leg of each mouse to its paired ‘control’ untreated limb (figure 3). This related assay of skin damage showed a FLASH effect only in the single fraction groups. All single dose groups demonstrated significant differences between UHDR and CDR groups of mice (*P* < 0.05). We determined that a single-dose UHDR delivery did not significantly affect the limb mobility of a mouse for 22 & 24 Gy doses, as compared to the same dose CDR delivery, and a small effect at 30 Gy dose. Again, as with the skin damage score assay, there was a monotonic increase in the magnitude of leg contracture loss with increasing dose.

## 4. Discussion

While there are a range of dosimetry parameters that can be modified in the FLASH effect (total dose D, average DR, instantaneous DR, dose per pulse, etc), one of the most pressing issues in FLASH research is to determine how to translate this to the norms of human radiotherapy, where fractionation is standard. The practical reality of trying to choose a reasonable dose fractionation scheme is essential to guide choices for translation to veterinary animal and human studies. It appears established that there is a minimum total dose required and a minimum average DR for observation of FLASH skin sparing (Bohlen *et al* 2022a, 2022b). However, to make the translation conform to standard techniques, ideally hypofractionation schemes might be used with UHDR to match conventional paradigms in radiotherapy. This was the motivation for this study design, and the dose escalation was used to test different ranges of minimum dose that could be used. The results appear quite conclusive that single fraction treatments were significant in observation of the FLASH effect, whereas three daily fractions eliminated this effect at similar BED values. This was seen in terms of both skin damage response assays as well as the leg contraction assay. The use of BED for comparison of single dose deliveries to hypofractionation is not ideal as BED calculations primarily focus on >3 fractions with BED values being calculated from the LQ model based on *α*/*β* values from CDR data often well below 10 Gy per fraction, with this in mind it is still believed to be more appropriate than simply comparing total dose values in various fractions. Ideally a formulation of BED using UHDR derived *α*/*β* values that could incorporate dose rate effects with fraction could be derived for both acute and late responding tissues. Further studies examining intermediate changes in dose rate and various steps in fractionation, similar to what Bohlen *et al* (2022a) described to create a pool of UHDR focused BED values to be used in future studies. In this study single dose deliveries, across all dose values, did more acute skin damage as compared to the fractionation data sets at equivalent CDR defined BEDs. This study focused on acute damage in skin, further tissues should be analyzed alongside long term radiation effects to fully catalog the effect of fractionations impact on the FLASH effect.

### 4.1. Effect of dose fractionation

In the fractionated treatments, three different doses were delivered on consecutive days to determine if this conventional design might preserve the FLASH skin sparing effect with UHDR delivery. The minimal dose per fraction (10 Gy) for this study was chosen in accordance with the currently used doses in veterinary RT for osteosarcoma treatments (Yu *et al* 2014, Kubicek *et al* 2016, Nolan *et al* 2020, Martin *et al* 2021). The dose escalation from this level was done to ensure that we captured the effect as a function of total dose per fraction as well.

The murine skin assays were chosen as well-established models for studying acute radiation effects (Velalopoulou *et al* 2021). Skin toxicity is now the primary target for ongoing FLASH translational studies (Gjaldbaek *et al* 2024, Mascia *et al* 2024, Sorensen *et al* 2024) and provides a useful, reasonably safe, first step to examine FLASH efficacy. This is also highly relevant for electron RT where skin is receiving the maximal prescribed dose. Additionally, following up on a limb mobility in parallel helps to have a secondary, related measure of acute skin damage. The assays showed a clear monotonic dose response and a clear FLASH sparing effect in the UHDR group in a single fractionation.

The results indicate that fractionation essentially nullifies the FLASH effect in acute skin response. While we observe the clear monotonic dose response from fractionated deliveries and that fractionation lowered the magnitude of skin damage in most cases, the tissue sparing effect of UHDR is no longer present. Although fractionated data is limited the use of split doses in recent studies show that splitting doses from single deliveries into equivalent doses delivered 2 min apart results in a significant reduction in the FLASH effect (Mascia *et al* 2023, Sorensen *et al* 2024). This highlights the need for further studies of fractionation effects in various tissues to understand how critical fractionation is to the sparing of UHDR RT.

However, it is important to note that the FLASH effect is clearly demonstrated in the single dose delivery, and the damage timeline observed matches the expected timeline of DNA damage in radiotherapy (Pottern 1985, Tavakkoli *et al* 2023, Gjaldbaek *et al* 2024). Independent of the mechanism, the timeline of damage for, indicating that both the CDR and UHDR dose rates appear to induce DNA damage related skin death, and both are generally believed to lead to recovery from the basal cell layer repair as described by Hopewell (Pottern 1985, Hopewell 1990, Joiner 2018). The kinetics are consistent with classic radiation damage seen for decades. This observation is important because the actual mechanism of FLASH sparing is still debated and this data is all consistent with that of DNA damage response. It is important to note that the UHDR deliveries not only result in the reduced damage at lower doses, but also demonstrate the skin recovery and healing within the follow-up timeline at even the highest doses for our visual skin assessment (figures 2(c) and (f)), while leg contracture shows little to no recovery (figures 3(c) and (f)).

**Figure 4.**
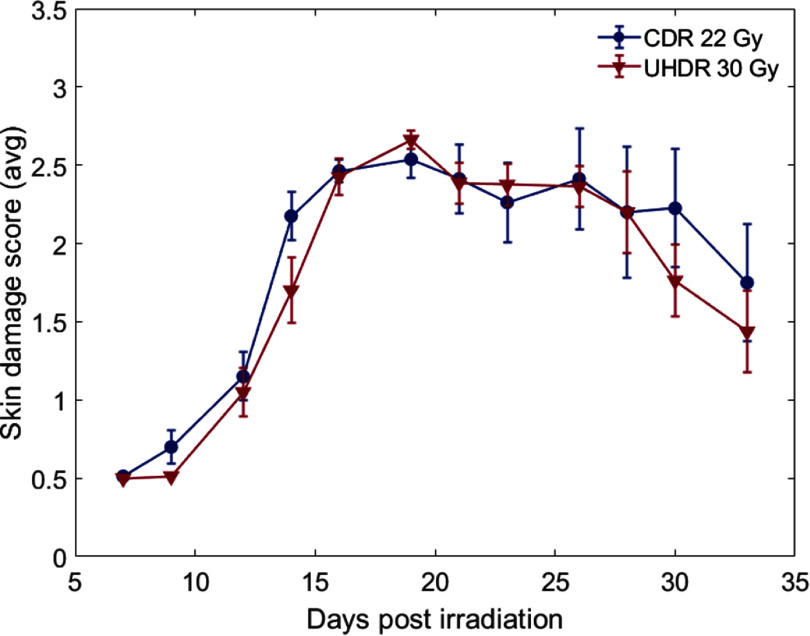
Demonstration of FLASH sparing effect matching damage response for increased D, with 30 Gy at UHDR versus 22 Gy at CDR. This corresponds to a FLASH modifying factor (FMF) factor of 0.73 (Bohlen *et al* 2022a).

From both blinded skin damage scoring and the unblinded leg contracture scoring we conclude that while the fractionation of the dose does not result in additional normal tissue sparing after UHDR, the single dose delivery produces a significant FLASH effect even at the doses as high as 30 Gy. UHDR dose delivery only provides the normal tissue sparing in the groups where full dose was applied in single delivery. Figure 4 shows that we obtain the same damage at the same biological effect after 22 Gy of CDR that appears in 30 Gy dose under UHDR. This corresponds to a electron FLASH modifying factor (FMF) (equation (1)) of 0.73 with a 95% confidence interval of 0.65–0.82 when comparing the maximal damage values. The FMF was is described by Bohlen *et al* (2022a).

\begin{equation*}{\text{FMF}} = \frac{{{D_{{\text{CDR}}}}}}{{{D_{{\text{UHDR}}}}}}{|_{{\text{isoeffect}}}}.\end{equation*}

### 4.2. Skin scoring system

A variety of published scoring systems are available for visual grading of skin damage in mouse models in radiation studies (Pogue *et al* 2024). Radiation induced acute skin damage has a well characterized timeline and intensity with damage beginning ∼10 d post treatment, peaking at ∼15–19 d post treatment, afterwards healing begins due to the survival of basal stem cells (Pottern 1985). The choice of an appropriate scoring system for the study design is critical to identifying the optimal dose range (Singers Sorensen *et al* 2022). Sorensen *et al* showed the most robust data set on FLASH RT in mouse skin and used a skin damage scoring scale that weighs higher damage observations such as fused toes and feet in the mice. This scoring scale was designed to show the highest magnitude of a FLASH effect, but these show an optimal FLASH sparing effect at doses 40–50 Gy and above. It is not entirely clear that the difference is between the two scoring systems, but in both studies the maximal flash effect was clearly seen at the lowest single dose group, in both assays of this study (Singers Sorensen *et al* 2022, Sorensen *et al* 2022, 2024).

Two notable difference between this study and studies by Sorensen *et al* is the volume of irradiated tissue, which is known to have a large effect on dose required to induce equivalent effects (Hopewell and Trott 2000, Joiner 2018), and the use of a different mouse strain (CDF1). Sorensen *et al* primarily irradiated feet, while in these studies the entire limb was targeted as demonstrated in figure 1. Although the difference between irradiation volumes in the order of cm’s this is crucial to mention as a mouse foot is ∼1–2 cm in length with the entire leg being roughly 3 cm in length. Studies on the volume effect in skin show that a field diameter of <2 cm in pigs and humans drastically increased the dose required for equivalent effects (Hopewell and Trott 2000). While data is limited in murine skin models due to their size it is robustly reported in other murine organs such as spinal cord (van der Kogel 1993). This volume effect in skin could result in an increase of dose required to induce equivalent effects detailing why the scoring system in this study saturates and hits maximal scoring metric i.e. 3.5, and are unable to resolve damage past)at ∼30 Gy in CDR while Sorensen *et al* sees no damage at this level ranking as 2.5, a moderate value on their scale. In comparing FMF values Sorensen *et al* show an FMF of ∼0.58 at doses of 25 Gy CDR and 39 Gy UHDR and in a complementary study Kristensen *et al* (2025) in electron FLASH saw a FMF of ∼0.65 at 29 Gy CDR and 45 UHDR. The higher FMF in these studies could further be attributed to the higher dose points used.

Comparison with other studies is complicated due to their focus on different endpoints, such as (i) time until a skin effect occurs (Tavakkoli *et al* 2023), (ii) inflammation (Rudigkeit *et al* 2024), or (iii) number of mice developing skin damage, as primary experimental endpoints (Mascia *et al* 2024). The skin scoring system used in this study was deliberately chosen to be sensitive to more subtle skin damage and saturates before intense skin effects such as fusion of toes or limbs and necrosis. This was felt to be the appropriate choice for a preliminary study that would be used to define the dose levels used in translation to a veterinary or clinical trial where severe effects are prohibited.

We believe that the difference in the radiation damage caused by both CDR and UHDR deliveries lies in the difference in the irradiated areas. While the majority of the past studies would focus on irradiating the foot, such precise choice of an irradiation area is not as easily achievable in the electron beam deliveries. Most of the past studies used a proton pencil beam to deliver CDR or UHDR doses, and the unique feature of the proton beam—Bragg peak—allows to use the pencil beam which can irradiate smaller areas more precisely. In our study we irradiated the whole hind leg together with the foot. Here, the dose-volume effect explains that the skin dose can be significantly reduced by limiting the impact dose to the target volume. We believe that the increased damage to the leg while little to no damage to the foot is explained by the dose impact on the larger area of the mouse skin.

Another key point of the estimating the radiation-induced skin damage in this study was the use of multiple trained observers, blinded to the study. The pictures of the mice irradiated legs were randomized for each follow-up day and then distributed among the observers. Afterwards, the data was averaged per mouse to allow for the better statistical results and less potential for observer bias. The use of the multiple estimators helped to minimize errors.

## 5. Conclusions

This work provides conclusive evidence needed about how the use of daily hypofractionation may affect observations of FLASH skin sparing. The data in the assays presented here were robust and both showed clear evidence of loss of the FLASH effect at all dose levels when three fractions were used instead of a single dose treatment, in acute skin damage of C57Bl/6j female mice. It seems likely that there is a minimum total dose required to see the FLASH effect benefit and that splitting up doses into fractions reduces this effect (Bohlen *et al* 2022a). Both visual skin damage and leg contracture assays had monotonic and predictable dose responses with dose escalation, in both of the single and fractionated groups. They also provided statistically significant differences in all single dose groups for CDR versus UHDR differences, as evidence of a clear FLASH effect. Given these results, it seems likely that next translation steps in FLASH skin research may focus more on single dose delivery in the range between 16 and 24 Gy to observe the FLASH effect in larger animals or humans. Our data is consistent with the idea that this range is where there is a minimum total dose at which the FLASH sparing effect occurs and identification of this minimum level should be established for each tissue type being investigated. These results are being utilized for design of a translational study in veterinary animals.

## Data Availability

All data that support the findings of this study are included within the article (and any supplementary information files).
